# Vitamin D_3_ supplementation as an adjunct in the management of childhood infectious diarrhea: a systematic review

**DOI:** 10.1186/s12879-023-08077-3

**Published:** 2023-03-14

**Authors:** Samuel N Uwaezuoke, Chioma L Odimegwu, Ngozi R Mbanefo, Chizoma I Eneh, Ijeoma O Arodiwe, Uzoamaka V Muoneke, Francis N Ogbuka, Chibuzo O Ndiokwelu, Anthony T Akwue

**Affiliations:** 1 Department of Pediatrics, The University of Nigeria Teaching Hospital Ituku-Ozalla Enugu, Enugu, Nigeria; 2 Department of Pediatrics, Enugu State University Teaching Hospital, Enugu, Nigeria; 3 Emergency Department, ASEER field Hospital, Mecca, Kingdom of Saudi Arabia

**Keywords:** Adjunct therapy, Childhood, Cholecalciferol, Infectious diarrhea, Innate immunity

## Abstract

**Background:**

Some studies have reported the possible role of vitamin D_3_ in ameliorating disease outcomes in childhood infectious diarrhea. However, findings about its effectiveness and the association of serum vitamin D levels with diarrhea risk appear inconsistent. We aimed to determine the efficacy of oral vitamin D_3_ as an adjunct in managing childhood infectious diarrhea and the relationship between vitamin D status and the disease.

**Methods:**

We searched the PubMed and Google Scholar electronic databases for relevant articles without limiting their year of publication. We selected primary studies that met the review’s inclusion criteria, screened their titles and abstracts, and removed duplicates. We extracted data items from selected studies using a structured data-extraction form. We conducted a quality assessment of randomized controlled trials (RCTs) and non-randomized studies with the Cochrane collaboration tool and the Newcastle Ottawa Scale, respectively. We assessed the strength of the relationship between serum vitamin D levels and diarrhea using the correlation model. We estimated the I^2^ and tau^2^ values to assess between-study heterogeneity.

**Results:**

Nine full-text articles were selected, consisting of one RCT, three cross-sectional studies, two cohort studies, two longitudinal/prospective studies, and one case-control study. A total of 5,545 participants were evaluated in the nine studies. Six non-randomized studies provided weak evidence of the relationship between vitamin D levels and diarrhea risk as there was no correlation between the two variables. The only RCT failed to demonstrate any beneficial role of vitamin D_3_ in reducing the risk of recurrent diarrhea. The calculated I^2^ and tau^2^ values of 86.5% and 0.03, respectively suggested a high between-study heterogeneity which precluded a meta-analysis of study results.

**Conclusion:**

Oral vitamin D_3_ may not be an effective adjunct in managing childhood infectious diarrhea. Additionally, the relationship between vitamin D status and infectious diarrhea appears weak. We recommend more adequately-powered RCTs to determine the effectiveness of vitamin D_3_ as an adjunct therapy in infectious diarrhea.

## Background

Diarrhea is one of the top-four infectious causes of childhood morbidity and mortality in tropical developing countries: the remainder comprising pneumonia, malaria, and human immunodeficiency virus (HIV)/acquired immune deficiency syndrome (AIDS) [1, 2]. The World Health Organization (WHO) statistics reveal the enormous health burden associated with childhood infectious diarrhea. For instance, it is the second leading cause of mortality in under-five children, as more than half a million succumb to diarrhea-related deaths yearly [3]. Also, there is global documentation of nearly 1.7 billion diarrhea cases annually [3]. The incidence rate is high in low-income countries (LICs), where each child under three years experiences three episodes of diarrhea on average every year [3]. Contaminated water from poor sanitary hygiene is a significant source of contracting the disease in these settings. Water contamination with fecal matter (due to the high rate of open defecation) constitutes a public health challenge. Thus, rotavirus and *Escherichia coli* are the most common etiologic agents of moderate-to-severe diarrhea in LICs [3].

There is a standard management protocol that mitigates the adverse consequences associated with childhood diarrhea. The protocol comprises supplemental zinc and low osmolarity oral rehydration solution (ORS) and has increased patients’ survival rates over the years [4–6]. Zinc is critical in modulating the host’s resistance to infectious agents and reducing diarrhea risk, severity, and duration [6]. Its precise mechanism of ameliorating diarrhea-related morbidity is largely unresolved. However, the micronutrient enhances the absorption of water and electrolytes, stimulates intestinal neo-epithelialization, and increases the levels of brush border enzymes [7]. Also, zinc promotes a better clearance of etiologic pathogens by increasing T lymphocytes and macrophage levels [7]. Thus, zinc deficiency negatively impacts the immune system’s maturation [8] and may explain why children with reduced serum zinc levels experience either severe diarrhea or higher episodes of diarrhea [9].

Similarly, vitamin A is another micronutrient considered an adjunct in treating childhood diarrhea. Oral vitamin A is associated with decreased incidence rates of diarrhea and its related mortality [10]. Nevertheless, there is no consensus yet on this beneficial effect. For instance, a randomized controlled trial (RCT) demonstrated that oral vitamin A supplementation did not affect the duration of diarrhea during an acute episode in well-nourished infants aged between 6 and 12 months [11].

Recently, there has been a renewed interest in using oral vitamin D_3_ to improve the outcomes of childhood infectious diarrhea. Given the pleiotropic nature of vitamin D, it modulates immunologic function: particularly the enhancement of innate immunity, such as the production of gut antimicrobial peptides [12–14]. Because of this link with enteric immunologic function, its role in infectious diarrheas is now a research subject. For instance, some investigators reported that low serum 25-hydroxyvitamin D level was associated with increased intensity of diarrhea and poor disease outcomes in Bulgarian toddlers [15]. Furthermore, a cohort study in Iranian children revealed a negative correlation between serum 25-hydroxyvitamin D level and acute bacterial diarrhea; thus, the authors suggested that vitamin D could be involved in the pathogenesis of diarrhea [16]. However, other researchers in Afghanistan noted that oral vitamin D_3_ failed to reduce the risk for recurrent diarrhea in a population of infants they studied [17].

We initiated this systematic review because of these inconsistent findings, focusing on controlled intervention studies that utilized vitamin D_3_ to prevent or treat infectious diarrheas and studies that evaluated the relationship between serum 25-hydroxyvitamin D levels and incident diarrhea in children. We thus aimed to determine the efficacy of oral vitamin D_3_ as an adjunct in managing childhood infectious diarrhea, and the relationship between vitamin D status and the disease. We conducted and reported the systematic review in conformity with the Preferred Reporting Items for Systematic reviews and Meta-analyses (PRISMA) guidelines [18].

## Methods

### Protocol and registration

There was no review protocol for the present systematic review.

### Literature search strategy

We searched the PubMed and Google Scholar electronic databases for relevant articles without limiting their year and language of publication. Based on the title of the systematic review, we used the following descriptors in PubMed in multiple combinations (as MeSH terms or not) with Boolean operators (AND/OR): (“cholecalciferol“[MeSH Terms] OR “cholecalciferol“[All Fields]) AND “childhood“[All Fields] AND (“dysentery“[MeSH Terms] OR “dysentery“[All Fields] OR (“infectious“[All Fields] AND “diarrhea“[All Fields]) OR “infectious diarrhea“[All Fields]). The date of the last search was 31 August 2022. We also used descriptors like ‘infectious diarrhea,’ ‘cholecalciferol,’ ‘childhood,’ and ‘adjunct therapy’ to search the Google Scholar database for related articles.

### Inclusion and exclusion criteria

We selected primary studies which met the inclusion criteria. These criteria include cohort studies or randomized controlled trials (RCTs) on human subjects, cross-sectional or case-control studies that evaluated the association of vitamin D status (serum 25-hydroxyvitamin D level or vitamin D-binding protein [DBP] level as a surrogate marker) in children with episodes of diarrhea, and full-text articles with these study designs published in or translated into the English language. Excluded articles comprised abstracts, reviews, editorials, commentaries, conference proceedings, and studies without primary data.

### Study selection

We screened the titles and abstracts of retrieved articles from the two electronic databases and independently assessed potentially eligible full-text articles for selection and inclusion in the final list of papers for review. We resolved possible disagreements on selected studies by consensus. We excluded duplicates and primary studies whose objectives were not in tandem with the aim of the present systematic review.

### Quality assessment

We assessed the methodological quality of each selected study using Newcastle-Ottawa Scale (NOS) [19] and Cochrane collaboration’s tool [20] for non-randomized studies and RCTs, respectively. The Newcastle Ottawa Scale consists of the following criteria for evaluating case-control or cross-sectional studies: ‘selection’ (maximum of 5 stars), ‘comparability’ (maximum of 2 stars), and ‘exposure/outcome’ (maximum of 3 stars). We rated the quality of each study high if the assigned score is ≥ 7 stars or low if the score is ≤ 7 stars. The Cochrane collaboration’s tool assesses the risk of bias in RCTs based on seven parameters: random sequence generation, allocation concealment, blinding of participants and personnel, blinding of outcome assessment, incomplete outcome data, selective reporting, and other biases. For each parameter, we adjudged a study as having a low risk of bias (+), high risk of bias (-), or unclear risk of bias (?).

### Data extraction and data items

We extracted the following data items from the selected articles using a structured data-extraction form: author’s name, year of publication, study setting, design, population, country of study, sample size, and patient demographics such as age and sex. Other extracted items were the pharmacologic interventions in the form of oral vitamin D_3_ or the estimation of serum 25-hydroxyvitamin D (the most reliable reflection of vitamin D level in the body) or DBP level (as a surrogate marker), and the assessment of diarrhea outcomes or incident disease in the study population. We also retrieved the risk of bias in the RCTs.

### Data synthesis

We qualitatively synthesized the extracted data to determine if there were differences in disease incidence or outcomes (between the intervention and the control groups) that had statistical significance. We also synthesized data on the serum 25-hydroxyvitamin D or DBP level (as a surrogate marker), to determine the strength of the relationship between vitamin D status and episodes of diarrhea. We assessed the strength of this relationship using the correlation model. We estimated the I^2^ and tau^2^ values to assess between-study heterogeneity, with a focus on the differences in the composition of the study populations. We did not conduct a quantitative synthesis (meta-analysis) of study results to provide an overall estimate of the effect of vitamin D status on diarrhea episodes because of differences in study design and endpoints and the estimated values of heterogeneity: I^2^ = 86.5%; p < 0.001; tau^2^ = 0.03 (indicating a high heterogeneity across studies). Also, we conducted a subgroup analysis to identify the possible factors responsible for this significant heterogeneity.

## Results

### Study selection

**Fig. 1:**
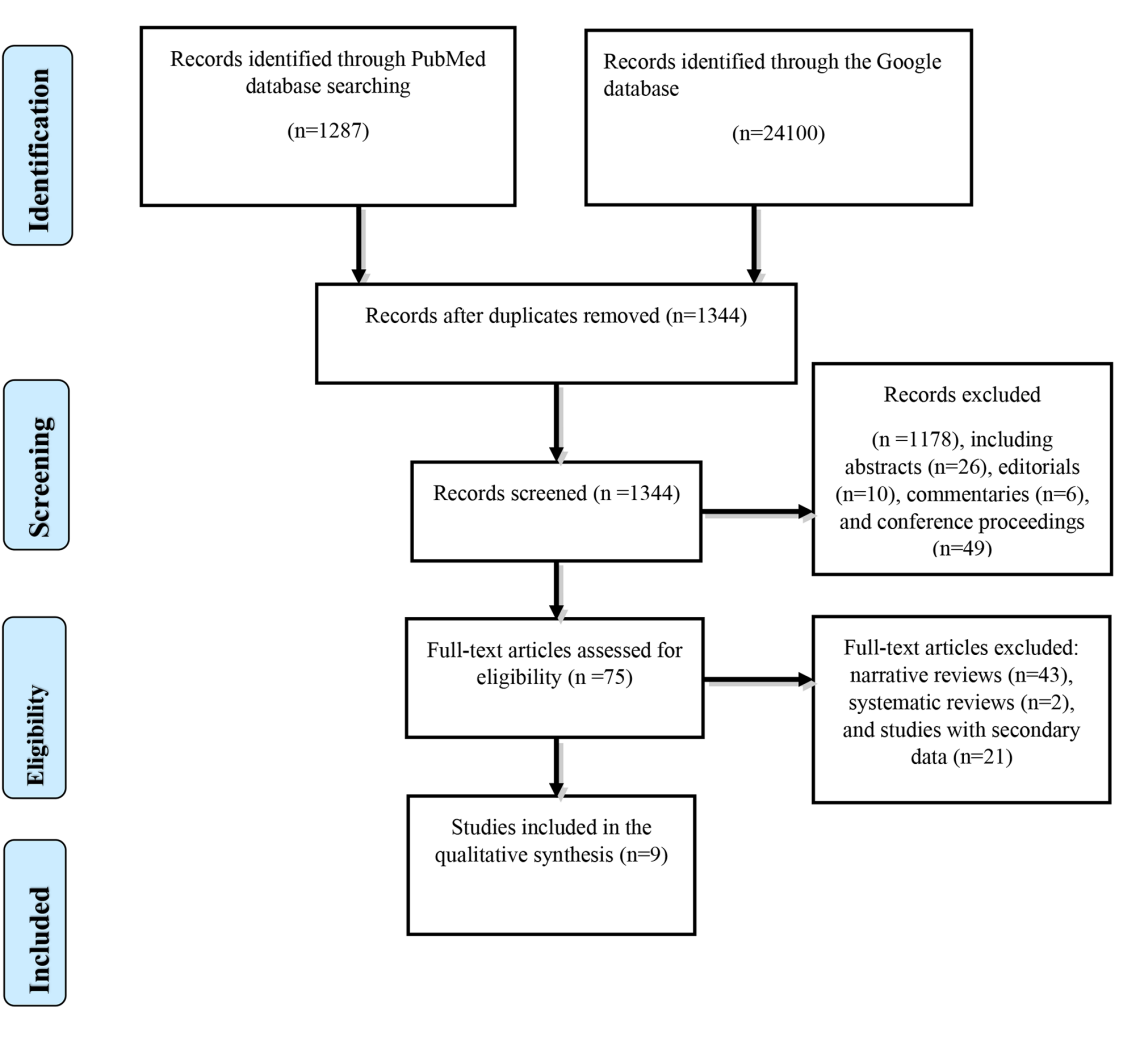
The Preferred Reporting Items for Systematic reviews and Meta-Analyses (PRISMA) algorithm for inclusion of studies on the relationship between vitamin D_3_ and childhood infectious diarrhea

The search of PubMed and Google Scholar databases yielded 1,287 and 24,100 articles, respectively: giving a total of 25,387. After removing duplicates and articles unrelated to the topic, the remaining papers were 1,344. Screening for their relevance to the present systematic review resulted in the exclusion of more records (n = 1178) - including abstracts (n = 26), editorials (n = 10), commentaries (n = 6), and conference proceedings (n = 49) - which scaled down the number of papers to 75. Following the assessment of the 75 full-text articles for eligibility, further exclusion of narrative reviews (n = 43), systematic review/meta-analysis (n = 2), and studies with secondary data (n = 21) yielded nine papers. We finally selected nine full-text articles for the present systematic review (Fig. 1).

### Study characteristics

As shown in Table 1, the nine selected full-text articles consist of one RCT [17], three cross-sectional studies [16, 22, 25], two cohort studies [15, 26], two longitudinal/prospective studies [23, 24], and one case-control study [21]. The countries of the studies are located in the Middle East [16, 22], Asia [17, 26], Europe [15, 25], South America [23, 24], and Africa [21]. One study was both hospital-and community-based [17], six studies were hospital-based [15, 16, 21–23, 25], while two were community-based [24, 26].

We evaluated 5,545 participants in the nine studies. They had a variable age and sex distribution. The majority of the participants were under-five children [15, 17, 21, 25, 26], and their ages ranged from 1 to 11 months [17], 12–42 months [15], 6–24 months [26], and 1–5 years [25]. In one study, the mean age was 17.01 ± 14.8 months [21]. Three studies that evaluated school-age children reported mean ages of 8.9 ± 1.6 years [23] and 8 ± 1.6 years [24], and an age range of 2 months-12 years [16]. One study reported equal sex distribution [17]. In contrast, three studies documented male predominance [16, 21, 22], while one noted a female predominance [24].

We applied star rating to items under parameters like selection and comparability of cases and controls (or cohorts) and assessment of outcome/ascertainment of exposure. In Table 2, the quality assessment of eight of the nine studies using the Newcastle-Ottawa Scale shows a star rating of > 7 (high quality) for six studies [15, 16, 21, 22, 25, 26]. We noted a rating of < 7 (low quality) for only two studies [23, 24]. These two studies were longitudinal in design and had no controls. Assessment parameters like selection and comparability of cases and controls (or cohorts) did not apply to these studies, thus precluding star rating on items under these parameters. The adjudged high-quality studies were cohort, cross-sectional, and case-control in design.

As shown in Table 2, the methodological quality of the only RCT [17], assessed with the Cochrane Collaboration tool, reveals a low risk of bias under five parameters: random sequence generation, allocation concealment, blinding of participants, and personnel, incomplete outcome data, and selective reporting. For instance, the authors documented evidence of randomization and masking in the trial. Unique identification numbers were individually randomized in fixed blocks of 20 to the vitamin D_3_ group, while the investigators applied randomization using a computer-generated list to the placebo group.

**Table 1:** Characteristics of studies that reported the relationship between vitamin D_3_ status and childhood infectious diarrhea

Study (first author’s name and year of publication)	Country of study	Study setting	Study population (sample size and age/sex distribution)	Study design
Aluisio et al. [17], 2013	Afghanistan	Five inner-city districts of Kabul/Passive surveillance center at Maiwind Teaching Hospital	-N = 3046 -1-11 months -Equal M/F distribution	Double-blind, placebo-controlled, randomized trial
Thornton et al. [23], 2013	Colombia	Hospital-based setting in Bogotá	-N = 475 -Mean (± SD) age: 8.9 ± 1.6 years	Longitudinal/Prospective study
Mileva et al. [15], 2014	Bulgaria	Department of Infectious Diseases, Medical University of Varna	-N = 77 (n = 30, group A patients^**†**^ & n = 47, group B patients^**‡**^) -12-42 months	Cohort study
Talachian et al. [22], 2015	Iran	Department of Pediatrics, Hazrat-e-Rasoul Akram Hospital, Tehran	-N = 50 (n = 25 with acute infectious diarrhea & n = 25 as controls) -6 months-15 years -Mean (± SD) age: 25.9 ± 25.6 months M/F ratio: 1.7:1 * & 1.5:1 ^**§**^	Cross-sectional study
Bucak et al. [25], 2016	Turkey	Department of Pediatrics, Adıyaman University School of Medicine, Adıyaman	-N = 137 (n = 70 with rotaviral diarrhea & n = 67 as healthy controls) -1-5 years	Cross-sectional study
Ahmed et al. [26], 2016	Bangladesh	Community-based setting in the urban community of Mirpur, Dhaka	-N = 912 (n = 446 normal-weight children & n = 466 underweight children) -6-24 months	Cohort study
Palframan et al. [24], 2018	Colombia	Community-based setting in the context of Bogotá School Children Cohort	-N = 540 -Mean ± SD age: 8 ± 1.6 years -M/F:48%/52%	Longitudinal study
Mahyar et al. [16], 2019	Iran	Qazvin Children Hospital, affiliated with Qazvin University of Medical Sciences (Qazvin, Iran)	-N = 120 (n = 60 with acute bacterial diarrhea & n = 60 as controls) -2 months-12 years -M/F: 63.3%/36.7% * & 51.6%/48.4%^**§**^	Cross-sectional study
Hassam et al. [21], 2019	Tanzania	Muhimbili National hospital, Dar es Salaam	-N = 188 under-five children (n = 47, cases n = 94, sick controls & n = 47, healthy controls) -Mean ± SD age: 17.01 ± 14.8 months -M/F: 70.2%/29.8% * & 53.2%/46.8%^**§**^	Unmatched case-control study

**M**, male **F**, female **SD**, standard deviation * Case group ^**§**^Control group ^**†**^Patients with risk factors for severe diarrhea ^**‡**^Patients without risk factors for severe diarrhea

**Table 2:** The methodological quality of the nine selected studies using the Newcastle-Ottawa Scale and Cochrane Collaboration tool

Study (Study design)	Selection (max. of 5 stars)	Comparability (max. of 2 stars)	Exposure/outcome (max. of 3 stars)	Total (ten stars)^†^	RSG *	AC *	BPP *	BOA *	IOD *	SR *	OB *
Thornton et al. [23] (Longitudinal/prospective study)	2 stars	-	1 star	3 stars	N/A	N/A	N/A	N/A	N/A	N/A	N/A
Mileva et al. [15] (Cohort study)	4 stars	1 star	3 stars	8 stars	N/A	N/A	N/A	N/A	N/A	N/A	N/A
Talachian et al. [22], (Cross-sectional study)	4 stars	1 star	2 stars	7 stars	N/A	N/A	N/A	N/A	N/A	N/A	N/A
Bucak et al. [25] (Cross-sectional study)	4 stars	2 stars	2 stars	8 stars	N/A	N/A	N/A	N/A	N/A	N/A	N/A
Ahmed et al. [26] (Cohort study)	3 stars	1 star	2 stars	7 stars	N/A	N/A	N/A	N/A	N/A	N/A	N/A
Palframan et al. [24], (Longitudinal study)	2 stars	-	1 star	3 stars	N/A	N/A	N/A	N/A	N/A	N/A	N/A
Mahyar et al. [16] (Cross-sectional study)	3 stars	2 stars	2 stars	7 stars	N/A	N/A	N/A	N/A	N/A	N/A	N/A
Hassam et al. [21] (Case-control study)	3 stars	2 stars	2 stars	7 stars	N/A	N/A	N/A	N/A	N/A	N/A	N/A
Aluisio et al. [17], (Randomized control trial)	N/A	N/A	N/A	N/A	**(+)**	**(+)**	**(+)**	**(?)**	**(+)**	**(+)**	**(?)**

^**†**^Total rating of ≥ 7 stars and < 7 stars suggests high methodological quality and low methodological quality, respectively

**N/A**, not applicable **max.**, maximum * Parameters of the Cochrane Collaboration tool for randomized control trials

**RSG**, Random Sequence Generation **AC**, Allocation Concealment **BPP**, Blinding of Participants and Personnel **BOA**, Blinding of Outcome Assessment **IOD**, Incomplete Outcome Data **SR**, Selective Reporting **OB**, Other Bias

**Key to the risk of bias assessment**: low risk of bias **(+)**, high risk of bias **(-)**, or unclear risk of bias **(?)**

The study personnel and participants’ families were blinded to the treatment group to which the participants were assigned. There was an unclear risk of bias under parameters like blinding of outcome assessment and other biases (such as attrition bias). Specifically, the evaluation of diarrheal outcome involved caregivers’ recall of defecation history based on the 24 h preceding each outcome assessment visit, whereas the estimation of 25-hydroxyvitamin D levels was based on samples collected from randomly selected subsets of participants [17]. Additionally, 82.3% of the participants after the trial remained in follow-up with no significant difference in attrition between the vitamin D_3_ and placebo arms.

### Study findings

Table 3 A and 3B summarize the key findings of the nine reviewed studies. In the RCT by Aluisio et al., the authors aimed to evaluate the effects of quarterly supplementation with 100 000 IU of vitamin D_3_ (cholecalciferol) on the risk for recurrent diarrheal illnesses among children [17]. They randomized 3046 infants who received either oral vitamin D_3_ (n = 1524) or placebo (n = 1522) at 3-month intervals and followed up for 18 months. The study endpoints were diarrhea episodes (based on the WHO definition of diarrhea of ≥ 3 loose/liquid stools in 24 h). They noted incidences of diarrheal episodes of 3.43 (95% CI, 3.28–3.59) and 3.59 per child-year (95% CI, 3.44–3.76) in the placebo and oral vitamin D_3_ arms, respectively. Furthermore, the authors observed no effect on the risk for recurrent diarrheal disease in either intention-to-treat or per-protocol analyses (Table 3 A). Thus, they concluded that quarterly supplementation with vitamin D_3_ conferred no reduction in the risk of recurrent diarrheal disease [17].

The longitudinal study by Thornton et al. investigated the association of vitamin D status with gastrointestinal and ear infections in school-age children [23]. The authors determined the baseline vitamin D status of randomly selected children (N = 475) by estimating their plasma 25-hydroxyvitamin D levels and followed them up for an academic year. Interestingly, they found that vitamin D deficiency was associated with increased rates of diarrhea with vomiting (adjusted incidence rate ratio: 2.05; 95% CI: 1.19, 3.53) and earache/discharge with fever (adjusted incidence rate ratio: 2.36; 95% CI: 1.26, 4.44). These findings suggest an inverse relationship between vitamin D status and gastrointestinal/ear infections (Table 3 A). In another longitudinal study by Palframan et al., the investigators evaluated the associations between vitamin D binding protein (DBP) and gastrointestinal/respiratory infections in 540 school-age children [24]. DBP is a surrogate marker of vitamin D. They also examined whether such associations could be mediated through 25-hydroxyvitamin D (Table 3B). Plasma DBP and 25 hydroxyvitamin D were estimated at participants’ enrolment, followed by daily documentation of the infectious morbidity symptoms during the school year. The study endpoints were the rates of gastrointestinal and respiratory morbidity (i.e., the number of days of diarrhea with vomiting, cough with fever, and earache/ear discharge with fever divided by the number of days of observation). The authors found that DBP was inversely associated with the rates of diarrhea with vomiting and earache/ear discharge with fever. However, DBP-morbidity associations were not mediated through 25-hydroxyvitamin D.

The two cohort studies by Mileva et al. [15] (Table 3 A) and Ahmed et al. [26] (Table 3B) reported divergent findings. The former aimed to determine the vitamin D status in toddlers with acute diarrhea and to assess its relationship with diarrhea severity. The authors assayed circulating 25-hydroxyvitamin D_3_ levels in two groups of patients: Group A, with risk factors for severe diarrhea (n = 30), and Group B, without risk factors (n = 47). Diarrhea severity (i.e., more than 20 diarrheal stools per day) was the study outcome. They noted that patients in Group A were vitamin-D insufficient (median = 53.63 nmol/L) compared to those in Group B (median = 66.09 nmol/L). Vitamin D deficiency (median = 49.20 nmol/L) occurred in children with severe diarrhea (> 20 diarrheal stools) compared to vitamin D status in children (median = 64.93 nmol/L) with less severe diarrhea [15]. On the other hand, Ahmed et al. evaluated the association of vitamin D status with diarrhea episodes caused by Enterotoxigenic *Escherichia coli* (ETEC), Enteropathogenic *Escherichia coli* (EPEC), and Enteroaggregative *Escherichia coli* (EAEC) among underweight and normal-weight children (after controlling for other micronutrients status and household/socio-economic variables). At the enrolment of 912 study participants (n = 446 normal-weight children and n = 466 underweight children), the authors determined their serum vitamin D and another micronutrient status and isolated and characterized the causative organisms in stool samples collected during a diarrheal episode. ETEC, EPEC, and EAEC in diarrheal stool samples tested during five months of follow-up constituted the study outcomes. They found that vitamin D status was not independently associated with the risk of incident ETEC, EPEC, and EAEC diarrhea in underweight children. However, insufficient vitamin D status and moderate-to-severe retinol deficiency were associated with 44% and 38% reduced risk of incident EAEC diarrhea among normal-weight children [26].

The three cross-sectional studies by Mahyar et al. [16] (Table 3B), Talachian et al. [22], and Bucak et al. [25] (Table 3 A) reported similar findings. The study by Mahyar et al. aimed to determine the correlation between serum 25-hydroxyvitamin D and acute bacterial diarrhea in children [16]. The researchers estimated serum 25-hydroxyvitamin D levels in children with diarrhea (n = 60) and the control group (n = 60). They observed a significant difference between the mean ± SD of 25-hydroxyvitamin D levels in children with acute bacterial diarrhea (19.3 ± 7.8 ng/ml) and the control group (22.4 ± 7.3 ng/ml). Talachian et al. compared the serum levels of zinc, vitamins A, and D in children with infectious diarrhea with a control group by measuring and comparing baseline serum vitamin A, 25-hydroxyvitamin D_3_, and zinc levels in 25 children admitted with acute diarrhea and 25 children without the infection [22]. They found significantly lower 25-hydroxyvitamin D_3_ levels in the diarrhea group but no significant difference in vitamin A and zinc levels between the diarrhea and the control groups. In their study, Bucak et al. also compared serum 25-hydroxyvitamin D_3_ levels of hospitalized preschool children with rotaviral diarrhea (n = 70) with healthy controls (n = 67) [25]. The study interventions involved measuring and comparing serum levels of 25-hydroxyvitamin D_3_, parathormone, calcium, phosphate, alkaline phosphatase, complete blood count parameters, and C-reactive protein of the preschool children with rotaviral diarrhea and the controls without the infection. Using serum levels of 25-hydroxyvitamin D_3_ as their study endpoint, they noted significant differences between the mean serum 25-hydroxyvitamin D_3_ levels (14.6 ± 8.7 ng/mL) of rotaviral diarrhea patients and healthy controls (29.06 ± 6.51 ng/mL).

Finally, the case-control study by Hassam et al. aimed to determine the association between vitamin D levels and diarrhea in children under five years old [21]. The authors estimated serum vitamin D levels in children with diarrhea (n = 47), sick controls (n = 94), and healthy controls (n = 47). They categorized vitamin D status as vitamin D sufficient, insufficient, or deficient. Association between vitamin D status and diarrhea was taken as the primary outcome, while associations between diarrhea and independent variables were the secondary outcome. Despite the high prevalence of vitamin D deficiency in the participants, sick controls were 3.2 times and 5.03 times more likely to be vitamin D deficient than healthy controls. They also found that children with vitamin D deficiency were less likely to have diarrhea than those without vitamin D deficiency (Table 3B).

**Table 3:** (A) Major findings of the studies reporting the relationship between vitamin D_3_ and childhood infectious diarrhea (studies published between 2013 and 2016)

Study (first author’s name and year of publication)	Study aims	Study interventions	Study outcomes/endpoints	Major findings
Aluisio et al. [17], 2013	-To assess the effects of quarterly supplementation with 100 000 IU of vitamin D_3_ (cholecalciferol) on children’s risk for recurrent diarrheal illnesses.	-Randomization of recruited infants to receive either oral vitamin D_3_ (n = 1524) or placebo (n = 1522) at 3-month intervals and followed for 18 months	-Diarrhea episodes *	-The incidences of diarrheal episodes of 3.43 (95% CI, 3.28–3.59) and 3.59 per child-year (95% CI, 3.44–3.76) in the placebo and intervention arms, respectively. -No effect on the risk for recurrent diarrheal disease in either intention-to-treat or per-protocol analyses
Thornton et al. [23], 2013	-To investigate the association of vitamin D status with gastrointestinal and ear infections in school-age children	-Measurement of plasma 25-hydroxy-vitamin D levels in a random sample of children (N = 475) to determine their baseline vitamin D status. They were followed up for an academic year	-Incidence rate ratios & 95% CI for days with diarrhea, vomiting, diarrhea with vomiting, cough with fever, and earache or discharge with fever. ^**†**^	-Vitamin D deficiency,^**‡**^ associated with increased rates of diarrhea with vomiting (adjusted incidence rate ratio: 2.05; 95% CI: 1.19, 3.53) and earache/discharge with fever (adjusted incidence rate ratio: 2.36; 95% CI: 1.26, 4.44)
Mileva et al. [15], 2014	-To determine the vitamin D status in toddlers with acute diarrhea and evaluate its relationship with diarrhea severity	-Assay of circulating 25-hydroxyvitamin D levels in two groups of patients: Group A, with risk factors for severe diarrhea (n = 30), and Group B, without risk factors (n = 47)	-Diarrhea severity^**§**^	-Patients in Group A were vitamin-D insufficient (median = 53.63 nmol/L), compared to those in Group B (median = 66.09 nmol/L). -Vitamin D deficiency (median = 49.20 nmol/L) was detected in children with severe diarrhea compared to vitamin D status in children (median = 64.93 nmol/L) with less severe diarrhea
Talachian et al. [22], 2015	-To compare the serum levels of zinc, vitamins A, and D in children with infectious diarrhea with a control group	-Measurement and comparison of baseline serum vitamin A, 25-hydroxyvitamin D, and zinc levels in 25 children admitted with acute diarrhea and 25 children without the infection	-Serum levels of 25-hydroxyvitamin D, vitamin A, and zinc	-Significantly lower 25-hydroxyvitamin D levels in the diarrhea group -No significant difference in the levels of vitamin A and zinc between diarrhea and control groups
Bucak et al. [25], 2016	-To compare serum 25-hydroxyvitamin D level of hospitalized preschool children with rotaviral diarrhea with that of healthy controls	-Measurement and comparison of serum levels of 25-hydroxyvitamin D, parathormone, calcium, phosphate, alkaline phosphatase, complete blood count parameters, and C-reactive protein of preschool children with rotaviral diarrhea and controls without the infection	-Serum levels of 25-hydroxyvitamin D	-Significant differences between the mean serum 25-hydroxyvitamin D levels of rotaviral diarrhea patients (14.6 ± 8.7 ng/mL) and healthy controls (29.06 ± 6.51 ng/mL).^**¶**^

* Based on the WHO definition of diarrhea (≥ 3 loose/liquid stools in 24 h) CI, confidence interval ^**†**^Estimates adjusted for child’s age, sex, and household socio-economic status ^**‡**^Vitamin D status classified according to 25 hydroxyvitamin D_3_ levels as deficient (< 50 nmol/L), insufficient (≥ 50 and < 75 nmol/L) or sufficient (≥ 75 nmol/L) ^**§**^Above 20 diarrheal stools were considered severe. ^**¶**^Serum 25-hydroxyvitamin D_3_ < 20 ng/mL was associated with rotaviral diarrhea

**N/B**: 1 ng/mL is equivalent to 2.5 nmol/L

**Table 3:** (B) Major findings of the studies reporting the relationship between vitamin D_3_ and childhood infectious diarrhea (studies published between 2016 and 2019)

Study (first author’s name and year of publication)	Study aims	Study interventions	Study outcomes/endpoints	Major findings
Ahmed et al. [26], 2016	-To evaluate the association of vitamin D status (controlling for other micronutrients status and household/socio-economic variables) with ETEC, EPEC, and EAEC diarrhea episodes among underweight and normal-weight children	-Determination of serum vitamin D and another micronutrient status at enrolment -Isolation and characterization of causative organisms in stool samples collected during a diarrheal episode	-ETEC, EPEC, and EAEC in diarrheal stool samples tested during five months of follow-up	-Vitamin D status was not independently associated with the risk of incident ETEC, EPEC, and EAEC diarrhea in underweight children, but moderate-to-severe retinol deficiency was associated with reduced risk for EPEC diarrhea (upon adjustment). -Insufficient vitamin D status and moderate-to-severe retinol deficiency were independently associated with 44% and 38% reduced risk of incident EAEC diarrhea, respectively, among normal-weight children
Palframan et al. [24], 2018	-To investigate the associations between DBP and infectious morbidity among school-age children * -To examine whether any associations between DBP and morbidity could be mediated through 25-hydroxyvitamin D	-Estimation of plasma DBP and 25-hydroxyvitamin D at enrolment of subjects -Daily documentation of infectious morbidity symptoms during the school year	-Rates of gastrointestinal and respiratory morbidity^**†**^	-DBP was inversely associated with the rates of diarrhea with vomiting and ear-ache/ear discharge with fever -DBP–morbidity associations were not mediated through 25-hydroxyvitamin D.
Mahyar et al. [16], 2019	-To determine the correlation between serum 25-hydroxyvitamin D and acute bacterial diarrhea in children	-Estimation of serum 25-hydroxyvitamin D levels in children with diarrhea and control group	-Vitamin D status of study participants	-Significant difference between the mean ± SD of 25-hydroxyvitamin D levels in the case group (19.3 ± 7.8 ng/ml) and control group (22.4 ± 7.3 ng/ml)
Hassam et al. [21], 2019	-To determine the association between vitamin D levels and diarrhea in under-five children.	-Estimation of serum vitamin D levels in children with diarrhea^**‡**^	-Association between vitamin D status and diarrhea (primary outcome) -Associations between diarrhea and independent variables (secondary outcome)	-Children with vitamin D deficiency were less likely to have diarrhea as compared to children without vitamin D deficiency

**ETEC**, Enterotoxigenic *Escherichia coli***EPEC**, Enteropathogenic *Escherichia coli***EAEC**, Enteroaggregative *Escherichia coli***DBP**, vitamin D-binding protein * Gastrointestinal and respiratory infections ^**†**^The number of days of diarrhea with vomiting, cough with fever and earache/ear discharge with fever divided by the number of days of observation ^**‡**^Categorized as vitamin D sufficient, insufficient or deficient

**N/B**: 1 ng/mL is equivalent to 2.5 nmol/L

**Fig. 2:**
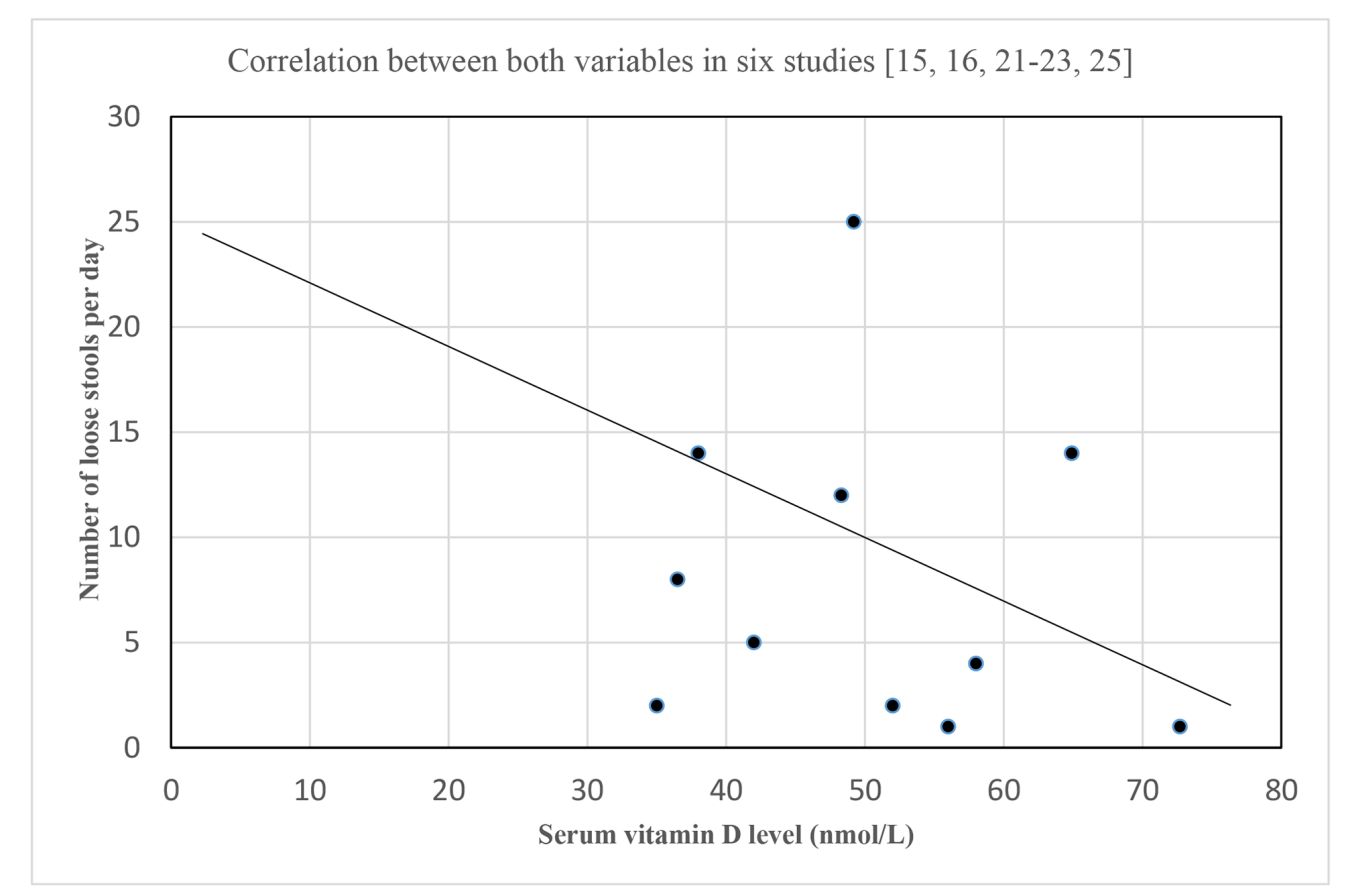
Scatter graph showing the nature of the correlation between vitamin D status and diarrhea in children

Given the inconsistencies noted in the findings of studies that evaluated the relationship between vitamin D status and diarrhea episodes, the strength of this relationship was assessed using a correlation graph. In Fig. 2, the scatter graph shows no significant correlation between the two variables in six studies [15, 16, 21–23, 25]. Assuming the covariance (X, Y) = 0 (from the pattern of the scatter graph), the Pearson correlation coefficient (*r*) was thus estimated to be 0, underscoring the absence of correlation between the two variables. All the six studies assessed vitamin D status by the quantitative estimation of serum vitamin D levels and adopted the conventional classification of vitamin D status: normal status (75–125 nmol/L), insufficiency (50–75 nmol/L), and deficiency (< 50 nmol/L).

### Subgroup analysis on association of vitamin D status with diarrhea risk

We categorized the study participants into age groups and analyzed the diarrhea risk with respect to their vitamin D status or vitamin D supplementation. In Table 4, vitamin D supplementation in infants showed no effect in reducing diarrhea risk. In the same age group, vitamin D deficiency and insufficiency were associated with increased and reduced diarrhea risk, respectively. Whereas vitamin D deficiency was associated with increased and decreased diarrhea risk in preschoolers, vitamin D insufficiency was associated with decreased diarrhea risk in the same age group. In school-aged children and adolescence, vitamin D deficiency was associated with increased diarrhea risk. Thus, the different outcomes of vitamin D status among infants and preschool-age children may partly explain the apparent non-correlation of vitamin D deficiency (independent variable) with diarrhea disease (dependent variable).

**Table 4:** Effect of age group on the relationship between vitamin D status and diarrhea risk

Age group	Vitamin D status/Vitamin D supplementation	Diarrhea risk	Study
-Infancy (1–12 months)	-Vitamin D supplementation -Vitamin D deficiency * -Vitamin D insufficiency^**†**^	-No effect in reducing risk -Increased diarrhea risk -Increased diarrhea risk -Decreased diarrhea risk^**§**^	-Aluisio et al. [17] -Talachian et al. [22] -Mahyar et al. [16] -Ahmed et al. [26]
-Preschool age (1–5 years)	-Vitamin D deficiency * -Vitamin D insufficiency ^**†**^	-Increased diarrhea risk -Increased diarrhea risk -Increased diarrhea risk -Increased diarrhea risk -Decreased diarrhea risk -Decreased diarrhea risk^**§**^	-Mileva et al. [15] -Talachian et al. [22] -Bucak et al. [25] -Mahyar et al. [16] -Hassam et al. [21] -Ahmed et al. [26]
-School age (6–12 years)	-Vitamin D deficiency *	-Increased diarrhea risk -Increased diarrhea risk -Increased diarrhea risk	-Thornton et al. [23] -Talachian et al. [22] -Mahyar et al. [16]
-Adolescence (13–18 years)	-Vitamin D deficiency *	-Increased diarrhea risk	-Talachian et al. [22]

* Serum vitamin D level < 50 nmol/L ^**†**^Serum vitamin D level = 50–75 nmol/L ^**§**^in normal-weight subjects

## Discussion

Some studies over the past decade report that oral vitamin D_3_ may ameliorate diarrhea-associated morbidity in children. Others have documented a possible correlation between low serum vitamin D levels and diarrhea episodes. Furthermore, there is a paucity of systematic reviews/meta-analyses on the role of vitamin D_3_ as a therapeutic adjunct in childhood infectious diarrhea. We initiated the present systematic review because of the lack of consensus in the literature.

In this review, we found that most of the studies indicate that vitamin D deficiency was associated with an increased risk of infectious diarrhea [15, 16, 22, 23, 25]. In contrast, DBP level was inversely related to rates of infectious diarrhea and respiratory infections [24]. These findings are consistent with several other studies that indicate a potential protective effect of vitamin D on infectious morbidity [27–32]. We suggest that these observations are predicated on the mechanistic actions of vitamin D_3_ in innate immunity. Calcitriol (active vitamin D_3_) levels are regulated by the antagonistic activities of the enzymes CYP27B1 and CYP24A1, which respectively increase and decrease calcitriol levels [33]. Once pathogens come in contact with the gut mucosa, they are recognized by toll-like receptors on macrophages resulting in the receptors’ immunologic activation: aiding intracellular expression of CYP27B1 and vitamin-D receptor (VDR) genes [34]. CYP27B1 produces calcitriol from adequate levels of 25-hydroxyvitamin D in the cytoplasmic matrix. The binding of calcitriol to VDR triggers the production of several endogenous antimicrobial peptides (AMPs), such as cathelicidin and β-defensin, which are widely expressed in the gastrointestinal tract [35, 36]. This calcitriol-VDR interaction also up-regulates nitric oxide (NO) synthase [37]. This pathophysiologic cascade of events explains why vitamin D deficiency may be associated with deranged innate immunity and thus increased susceptibility to intracellular pathogens etiologically linked to diarrhea. Whereas AMPs inhibit bacterial, viral, and fungal infections [38, 39], NO synthase complements bactericidal activity by up-regulating the oxidative burst in macrophages [40]. The clinical-practice implication for this finding is that improving the vitamin D status of children can serve as an ‘immunologic boost’ for them to withstand infectious diarrheas.

We also found that the only interventional study in our systematic review failed to demonstrate any beneficial role of vitamin D_3_ supplementation in reducing diarrhea morbidity [17]. Three-monthly supplementation of high-dose vitamin D_3_ (100,000 IU) did not confer protection against the risk of recurrent diarrhea. Similarly, an observational analytical study of two cohorts (underweight and normal-weight children) showed no relationship between vitamin D status and the risk of incident ETEC, EPEC, and EAEC diarrhea in underweight children [26]. However, the investigators noted that vitamin D insufficiency was associated with a reduced risk of incident EAEC diarrhea in children with normal weight [26]. Again, a case-control study observed that serum vitamin D levels were not explicitly associated with diarrhea in a population of under-five children [21]. These findings are in tandem with those of a previous systematic review of four trials which did not establish apparent differences between vitamin D-supplemented and-unsupplemented children regarding episodes of diarrhea [41]. The review concluded that vitamin D supplementation was not beneficial in reducing the incidence of childhood diarrhea. Although these observations are inconsistent with the findings of the previously-mentioned related studies [15, 16, 22–25, 27–32], some unidentified factors can explain this disparity. Our subgroup analysis identified age group as a possible factor. Age categorization on vitamin D status and diarrhea risk revealed divergent study outcomes in infants and preschoolers unlike in school-age and adolescent children. Specifically, vitamin D insufficiency was associated with decreased diarrhea risk in infancy and preschool age group. In contrast, vitamin D deficiency was associated with both increased and reduced diarrhea risk in the same age groups. Although the reason for these heterogeneous outcomes is not clear, we speculate that the age-related changes in the gut microbiota may be contributory. The diversity of gut microbiota is higher in adulthood than in childhood although interpersonal differences are higher in the latter than in the former [42]. Again, the gut microbiota assumes adult-like configuration during the first three years of life by which time the gut epithelium and mucosal barrier that it secretes provides a barrier against pathogenic micro-organisms [43, 44]. Dietary alteration may lead to changes in both the composition and diversity of gut microbiota [45]. For instance, formula feeding (and other factors like antibiotic use and caesarean section) may disrupt the composition of the gut microbiota [46]. In fact, the gut microbiota of formula-fed infants are more diverse than those of their breastfed counterparts [47], while children treated with antibiotics have less stable and less diverse flora [48]. Interestingly, some authors report that with age and in obesity, the metabolic activation of vitamin D_3_ (with the production of calcitriol) is reduced by hepatic steatosis and dysbiosis of the microbiota [49, 50]. The activation process by 25-hydroxylation occurs in the liver via the cytochrome P450 system and in the gut microbiome [51]. Thus, the reduced diarrhea risk reported among vitamin D-insufficient under-five children may be attributed to the protective effect of the diverse composition of their gut microbiota. On the other hand, the increased diarrhea risk noted among their vitamin D-deficient cohorts may be due to the reduced bioavailability of calcitriol. Decreased calcitriol levels follow poor vitamin D activation as a result of dysbiosis of the gut microbiota. The hypothesis appears validated by the fact that the study that observed the association of vitamin D insufficiency with decreased diarrhea risk reported this finding among normal-weight children [26]. Given the less mature and less diverse gut microbiota in malnourished than in normal-weight children [52, 53], it is not surprising that the latter’s gut microbiota composition could have been protective against diarrhea pathogens.

Although some authors suggest that a strong relationship between vitamin D status and diarrhea does exist, it may be masked by several other variables identified in interventional studies [54]. Firstly, the serum level of 25-hydroxyvitamin D required for calcium homeostasis and innate immunity varies. While there are existing standard recommendations of daily vitamin D needed to achieve calcium homeostasis, it is still challenging to predict the dose and duration of vitamin D that would optimize its non-calcemic or immunologic effects [55]. Although vitamin D administered in different frequencies (i.e., daily, weekly, or monthly) can maintain similar serum levels of 25-hydroxyvitamin D over an equivalent time frame [56], there is a strong possibility that poor adherence with daily vitamin D administration may result in insufficient vitamin D levels and suboptimal effects. Worse still, some children’s pre-morbid vitamin D status in some settings is deficient. For instance, a recent systematic review and meta-analysis comparing the pooled prevalence of vitamin D deficiency among poor and sick children in sub-Saharan Africa revealed a higher prevalence among healthy children [57]. Again, administering vitamin D_2_ (ergocalciferol) is adjudged less effective than vitamin D_3_ (cholecalciferol) at raising the serum levels of 25-hydroxyvitamin D [58]. Thus, differences in dosing strategies and the type of vitamin D may contribute to the disparities in the outcomes of trials on its effectiveness in childhood infectious diarrhea. Secondly, genetic variations of DBP (the major carrier protein for serum 25-hydroxyvitamin D) may play a role in the inconsistencies in study findings [59]. Some authors report that DBP polymorphisms may determine the amount of bioavailable serum 25-hydroxyvitamin D and therefore be more reflective of actual vitamin D status than total serum 25-hydroxyvitamin D [60]. Interestingly, in one of the studies evaluated in the present systematic review, DBP was inversely associated with gastrointestinal and respiratory infections, whereas these morbidity associations were not mediated through 25-hydroxyvitamin D [24]. Likely, these genetic variations could also mask the effects of vitamin D in some populations [54]. Finally, baseline 25-hydroxyvitamin level and VDR polymorphisms in study participants are also possible contributors to the disparities in the present review’s findings. The effectiveness of vitamin D in deficient subjects may be partly related to the inverse relationship between baseline 25-hydroxyvitamin D level and response to vitamin D administration [58]. Baseline vitamin D- sufficient individuals achieve a lesser elevation in 25-hydroxyvitamin D level than their deficient counterparts receiving vitamin D supplementation. Thus, studies with participants whose vitamin D status falls outside the range where the effects on infectious outcomes are obtainable may fail to show an improvement following supplementation [54]. Furthermore, some investigators have demonstrated that variants of VDR can affect response to vitamin D supplementation [61]. Their observation underscores the fact that VDR polymorphisms can also explain the inconsistent findings regarding the effectiveness of vitamin D supplementation as a therapeutic adjunct in infectious diarrheas.

The present systematic review has some limitations. The high between-study heterogeneity across the included studies precluded a quantitative synthesis (meta-analysis) of the overall effect of the study results. Additionally, most of our selected studies were non-interventional in nature, as there was no direct assessment of the impact of vitamin D supplementation on serum 25-hydroxyvitamin D levels. The studies evaluated relationships between participants’ vitamin D status and diarrhea morbidity outcomes. The high prevalence of vitamin D deficiency among healthy children in some settings [57] may be a confounder to the association of vitamin D status with infectious morbidity. Thus, non-recognition of this confounding variable will affect the generalizability of the study findings linking vitamin D deficiency with increased diarrhea risk.

## Conclusion

This systematic review has shown that vitamin D supplementation is not effective in reducing the risk of childhood infectious diarrhea. Although the association of vitamin D deficiency with infectious diarrhea risk (as demonstrated in three cross-sectional studies [16, 22, 25], one cohort study [15], and one longitudinal study [23]) suggested a possible relationship between vitamin D status and risk of gastrointestinal infections, another longitudinal study [24], one cohort study [26], and the case-control study [21] reported contrary findings. Over all, evaluating the strength of this relationship by correlation model showed a weak association between the two variables in six of the non-randomized studies [15, 16, 21–23, 25]. Nevertheless, the possibility of a strong relationship is supported by the well-documented role of calcitriol in innate immunity. When this non-calcemic action is attenuated, gut AMPs are not produced, resulting in the risk of infectious diarrhea. For future research direction, we recommend more adequately-powered RCTs on oral vitamin D’s role in reducing diarrhea risk. Such interventional studies should also control for potential confounding variables in the study population such as age group, DBP and VDR polymorphisms.

## Data Availability

The datasets generated and analyzed during the current study are not publicly available due to the authors’ decision but are available from the corresponding author on reasonable request.
